# Significance of 5-ALA-Guided Fluorescence in Resection of Invasive Intracranial Meningiomas: Findings from a Prospective Clinical Study

**DOI:** 10.3390/cancers17071191

**Published:** 2025-03-31

**Authors:** Masahide Matsuda, Narushi Sugii, Noriaki Sakamoto, Akinari Yamano, Eiichi Ishikawa

**Affiliations:** Department of Neurosurgery, Institute of Medicine, University of Tsukuba, Tsukuba 305-8575, Japan; narushi-sugii@md.tsukuba.ac.jp (N.S.); n.sakamoto@md.tsukuba.ac.jp (N.S.); yamano.akinari.yf@ms.hosp.tsukuba.ac.jp (A.Y.); e-ishikawa@md.tsukuba.ac.jp (E.I.)

**Keywords:** meningioma, invasion, 5-ALA, fluorescence, photodynamic diagnosis, prospective, clinical study

## Abstract

Background: In cases of intracranial meningiomas invading into surrounding tissues, determining the resection boundary can be challenging and often makes complete resection difficult. In such situations, the introduction of novel intraoperative techniques to identify infiltrative tumor components is desirable to improve the extent of tumor resection. Methods: A prospective clinical study was conducted on patients with intracranial meningiomas suspected of infiltration into the surrounding tissues. After completing the tumor resection under conventional white-light microscopy, intraoperative fluorescence diagnosis using 5-aminolevulinic acid (5-ALA) was performed to determine whether additional resection of the unintended residual tumor was feasible. Results: Intraoperative fluorescence diagnosis enabled additional resection of the residual tumor in 38.5% of the 13 enrolled cases and 45.5% of the 11 cases in which the tumor exhibited fluorescence positivity. Among the additional resected specimens, tumor infiltration was observed in all fluorescence-positive lesions of the bone and dura mater, whereas tumor cells were detected in only 33.3% of the fluorescence-positive areas in the adjacent brain parenchyma. Conclusions: Intraoperative fluorescence diagnosis using 5-ALA enhanced the extent of the resection of invasive meningiomas. Future large-scale studies are warranted to determine whether 5-ALA fluorescence diagnosis contributes to reducing tumor recurrence and improving overall survival in patients with invasive intracranial meningiomas.

## Simple Summary

Intracranial meningiomas typically have well-defined boundaries with the surrounding tissues, making the identification of resection margins relatively straightforward. However, some meningiomas exhibit invasive properties, leading to residual tumors after resection. Although intraoperative fluorescence diagnosis using 5-aminolevulinic acid (5-ALA), which is already widely utilized in malignant glioma surgery, has been suggested as a potential tool for visualizing invasive tumor components in intracranial meningiomas, its effectiveness in improving the extent of resection compared to that of conventional white-light surgery remains unclear. This prospective clinical trial targeted intracranial meningiomas suspected for the invasion of surrounding tissues. By applying intraoperative fluorescence diagnosis using 5-ALA after completing tumor resection under white light, this study demonstrated that fluorescence-guided surgery can improve the extent of resection of infiltrative tumor areas.

## 1. Introduction

Meningiomas are among the most common brain tumors, accounting for approximately 41% of all primary brain tumors [1]. Surgical resection plays a critical role in the treatment of meningiomas, and the extent of resection has been widely recognized to influence postoperative recurrence and overall survival [2,3,4,5]. In the resection of intracranial meningiomas, the tumor is typically well-demarcated from the surrounding tissue, and complete resection is generally considered the goal unless the tumor is adherent to critical blood vessels or cranial nerves [6,7]. However, in cases where the tumor infiltrates the brain tissue, skull, or soft tissue, or when the dura mater is diffusely thickened, determining the resection boundary of the tumor can be challenging, making complete resection difficult. In such cases, the introduction of new techniques that can identify infiltrative tumor tissues during surgery is desirable to improve the extent of resection.

In recent years, intraoperative fluorescence diagnosis using 5-aminolevulinic acid (5-ALA) has become widely recognized for its utility in detecting infiltrating tumor cells in malignant gliomas, leading to improved surgical resection [8,9,10,11]. Furthermore, in glioblastoma, it has been suggested that tumor fluorescence intensity may be associated with molecular profiles such as IDH1/2 mutations [12]. In meningiomas, 5-ALA is taken up by tumor cells more than normal cells due to differences in cellular metabolism and enzymes, resulting in the accumulation of protoporphyrin IX (PpIX) within the tumor [13,14]. Additionally, PpIX fluorescence is observed in tumor masses during surgery [15,16]. Therefore, the use of 5-ALA for intraoperative fluorescence diagnosis may be effective in identifying invasive tumor components in meningiomas that infiltrate the surrounding tissues. However, the precise role of PpIX fluorescence guidance in intracranial meningioma resection has not yet been established. Only one retrospective study has evaluated whether 5-ALA can improve the extent of surgical resection of intracranial meningiomas [17]. Additionally, systematic investigations of how 5-ALA can detect tumor infiltration into specific surrounding tissues are lacking.

The objective of this prospective study was to determine whether intraoperative fluorescence guidance with 5-ALA improves the degree of resection compared to surgery using only white light in intracranial meningiomas suspected of invasion into surrounding tissues based on preoperative imaging. Additionally, we analyzed the accuracy of identifying tumor infiltration in specific tissues, such as the brain, bone, and dura mater, using fluorescence diagnosis.

## 2. Materials and Methods

### 2.1. Study Design and Participants

This prospective study was conducted at the University of Tsukuba Hospital between June 2021 and December 2024. This study was approved by the Clinical Research Review Board of the University of Tsukuba (TCRB21-001/approved on 7 June 2021). Written informed consent was obtained from all patients. This study was registered in the Japan Registry of Clinical Trials (number jRCTs: 031210158).

The eligibility criteria for this study included individuals aged ≥20 years who were scheduled to undergo maximal surgical resection of a meningioma, those with suspected recurrent meningioma, and those with suspected primary meningioma exhibiting specific preoperative magnetic resonance imaging (MRI) findings. The signs suggestive of tumor infiltration into the surrounding tissues in cases of primary meningiomas were suspected invasion into the adjacent brain tissue or the skull, presence of hyperostosis or bone destruction in the skull, tumor progression beyond the skull, significant peritumoral brain edema, strong cystic degeneration within or around the tumor, irregularity of the tumor boundary, and the presence of microscopic lesions around the tumor.

### 2.2. Procedures

Prior to meningioma resection surgery, 5-ALA (20 mg/kg) dissolved in water was orally administered three hours (2–4 h) before anesthesia induction. First, tumor resection was performed under white light as the standard surgical procedure. Standard surgery was concluded when the surgeon determined the absence of residual tumors in the resectable areas. Factors determining unresectability included invasion into the venous sinus, adhesion to well-developed cortical veins, adhesion to eloquent brains, adhesion to major arteries, and adhesion to the cranial nerves. At this point, the surgeon determined the presence of any residual tumor that could not be resected because of adhesion to critical structures. Subsequently, excitation light was applied to the resected tumor and surgical field for fluorescence observation using a surgical microscope (KINEVO 900, Carl Zeiss AG, Oberkochen, Germany, or OH6, Leica Microsystems GmbH, Wetzlar, Germany) or a 4 K-3D exoscope (ORBEYE, Olympus, Tokyo, Japan). The fluorescence intensity of the tumor mass was observed and semi-quantitatively evaluated using three grades: strong, vague, and none. Next, the resection cavity was carefully examined for fluorescence-positive lesions. If fluorescence positivity was observed in areas other than those already declared unresectable owing to adhesion, additional resection was performed, if possible, and the tissue was submitted for pathological examination. The presence of tumor cells in the specimens that underwent additional resection was determined based on the pathological diagnosis.

### 2.3. Outcomes

The primary endpoint of this study was the proportion of cases in which additional tumor resection was achieved based on fluorescence diagnosis. Secondary endpoints included the fluorescence positivity rate of the tumor itself and the tumor detection rate in additionally resected specimens based on fluorescence diagnosis.

## 3. Results

The demographics and tumor characteristics of the 13 patients are summarized in Table 1. The study population included four men and nine women with an age range of 42–76 years and a median age of 66 years. Seven cases were newly diagnosed, whereas six (approximately half) were recurrent meningiomas. The most common tumor location was the convexity (four cases), followed by the sphenoid ridge and spheno-orbital region (two cases each). Other locations included the parasagittal region, olfactory groove, planum sphenoidale, middle fossa, and trigone of the lateral ventricle, with one case each. The histopathological diagnoses, determined without considering the information from fluorescence-positive additionally resected specimens, revealed that 10 cases (76.9%) were classified as World Health Organization (WHO) Grade 1, comprising meningothelial (six cases), transitional (three cases), and angiomatous (one case) subtypes. Two cases were atypical meningiomas (WHO Grade 2), and one was an anaplastic meningioma (WHO Grade 3).

Preoperative imaging findings suggestive of tumor invasiveness are detailed below. On head CT, skull invasion was suspected in six cases; hyperostosis, three; and, bone destruction, five (with overlapping findings). On head MRI, brain invasion was suspected in one case; extracranial tumor extension, seven; significant peritumoral brain edema, eight; marked cystic degeneration within or around the tumor, three; irregular tumor boundaries, 3; and microscopic lesions surrounding the tumor, two (with overlaps among the findings).

**Figure 1:**
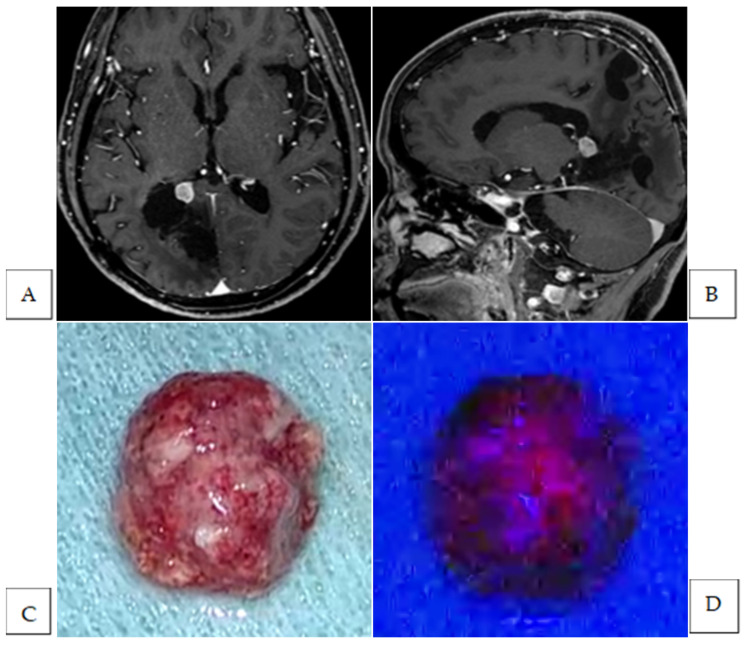

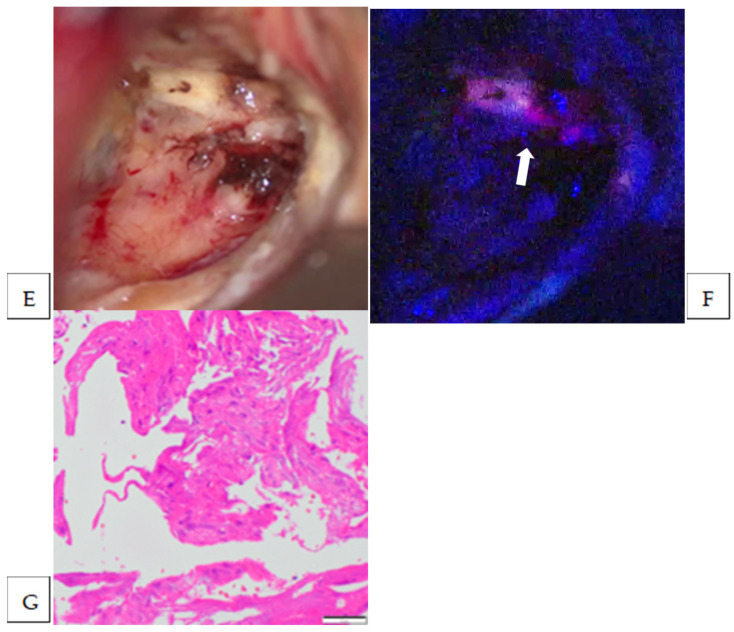
Preoperative Gd-enhanced T1-weighted axial (**A**) and sagittal (**B**) images of a recurrent trigone meningioma. Intraoperative photographs of the tumor mass under white (**C**) and blue light (**D**). The tumor mass exhibited strong fluorescence (**D**). Intraoperative photographs of the resection cavity after standard tumor resection under white light (**E**) and blue light (**F**). Fluorescence-positive lesions were also observed in the brain parenchyma. The hematoxylin and eosin-stained image of the additional resected fluorescence-positive specimen showing the presence of tumor cells (magnification, ×200) (**G**). The white arrow indicates a fluorescence-positive lesion.

**Figure 2:**
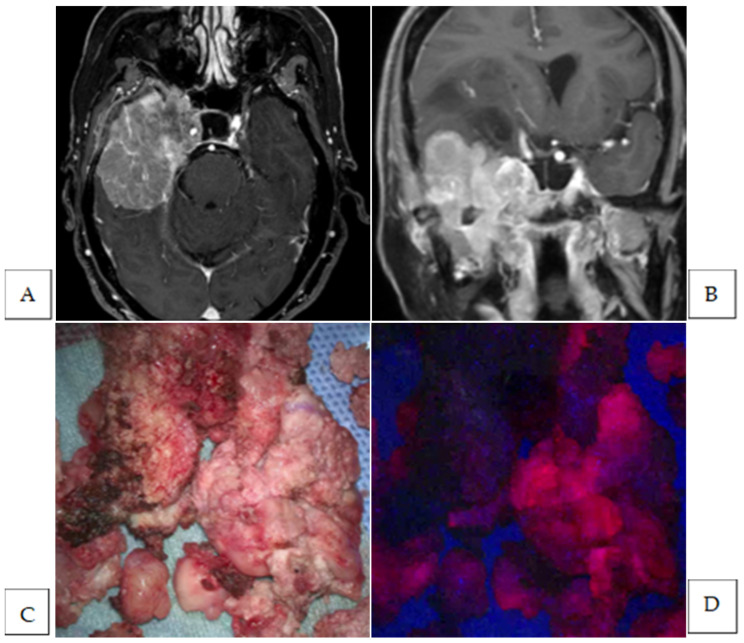

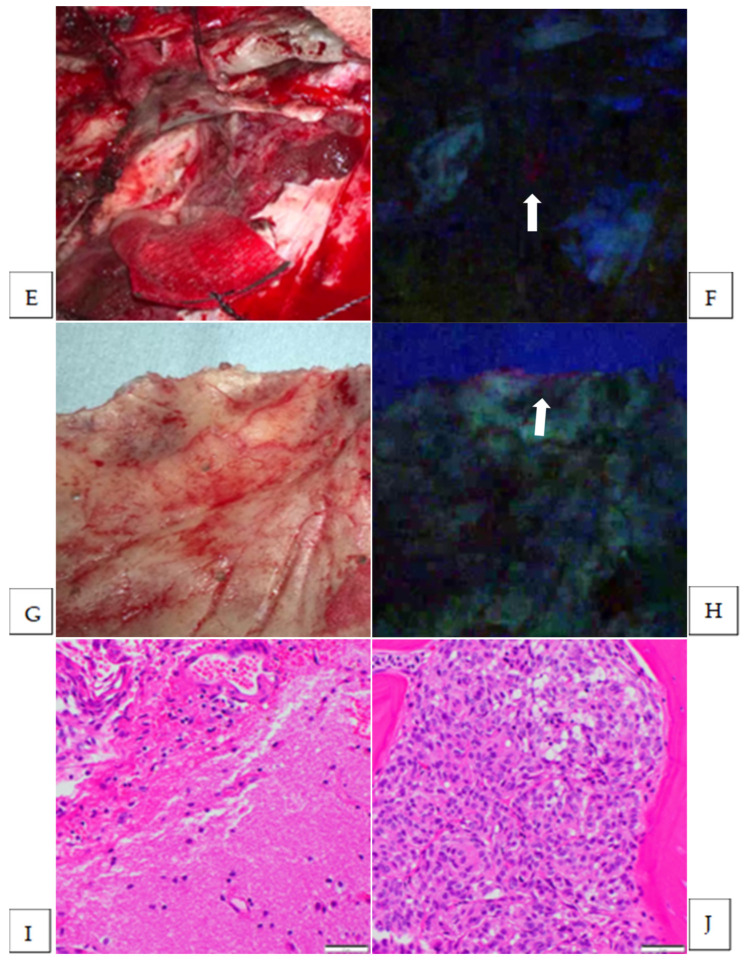
Preoperative Gd-enhanced T1-weighted axial image (**A**) and coronal image (**B**) of a middle fossa meningioma. Intraoperative photograph of the tumor mass under white-light (**C**) and under blue light (**D**). The tumor mass exhibits strong fluorescence (**D**). Intraoperative photograph of the resection cavity after standard tumor resection under white-light (**E**) and under blue light (**F**). Fluorescence-positive lesion is observed in the brain parenchyma (**F**). Intraoperative photograph of the bone flap after drilling out bone suspected of tumor invasion under white light (**G**) and under blue light (**H**). Fluorescence-positive lesion is observed in the bone flap (**H**). HE-stained image of the additional resected fluorescence-positive specimen from the brain parenchyma showing no tumor cells (**I**) and a fluorescence-positive specimen from the bone flap showing the presence of tumor cells (Magnification: ×200) (**J**). The white arrow indicates the fluorescence-positive lesion.

**Figure 3:**
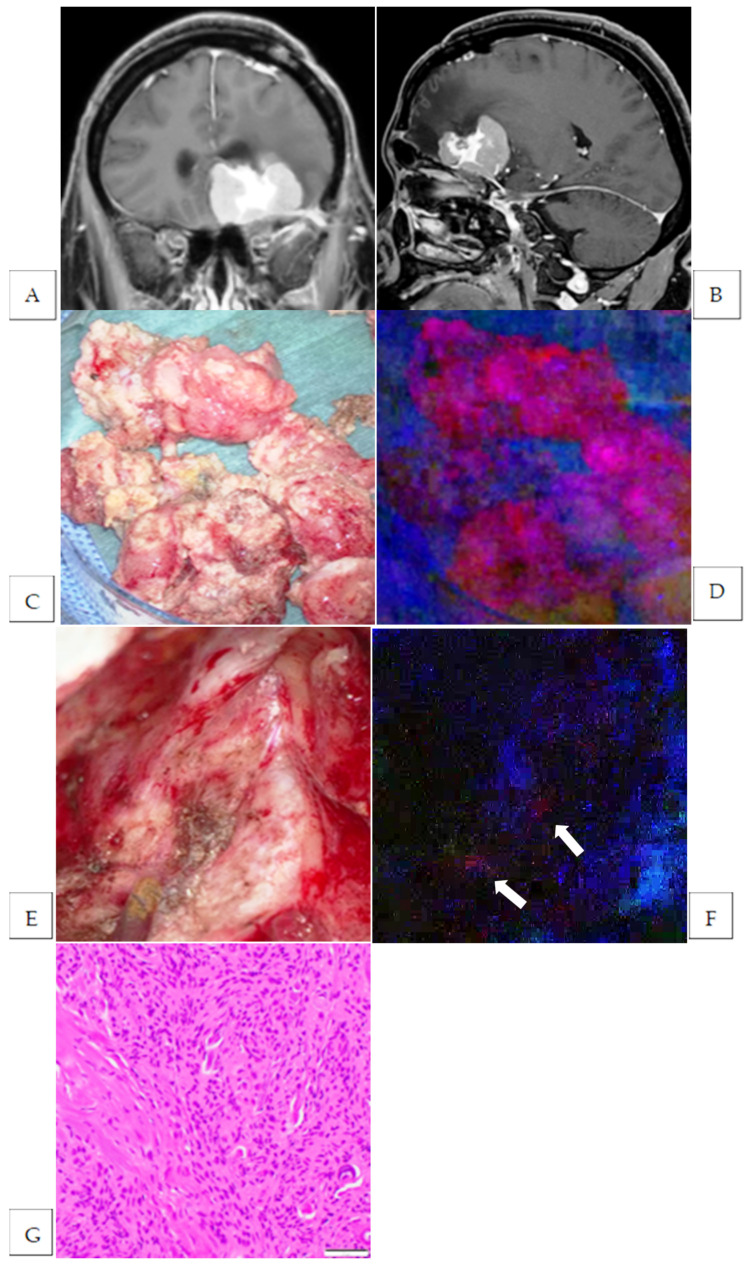
Preoperative Gd-enhanced T1-weighted coronal image (**A**) and sagittal image (**B**) of a recurrent planum sphenoidale meningioma. Intraoperative photograph of the tumor mass under conventional white light (**C**) and under blue light (**D**). The tumor mass exhibits strong fluorescence (**D**). Intraoperative photograph of the resection cavity after standard tumor resection under white light (**E**) and under blue light (**F**). Fluorescence-positive lesion is observed in the dura matter. HE-stained image of the additional resected fluorescence-positive specimen showing the presence of tumor cells (Magnification: ×200) (**G**). The white arrow indicates the fluorescence-positive lesion.

5-aminolevulinic acid was administered as planned in all 13 patients, and the fluorescence positivity of the resected tumor mass was observed in 11 patients (84.6%) (Table 2). The fluorescence intensity was strong in nine cases and vague in two cases. Unexpected residual fluorescence-positive lesions, other than those previously declared as unresectable before fluorescence observation, were identified in nine of the eleven fluorescence-positive cases (81.8%). Except for one fluorescence-positive lesion at the dural entry point of the superficial middle cerebral vein in the case of a middle fossa meningioma, all suspected residual lesions were located in resectable areas, and additional resection was performed for all these lesions. Residual fluorescence-positive lesions were located in the brain parenchyma (seven cases, nine sites), bone (two cases, three sites), and dura mater (three cases, four sites). Among the nine cases, excluding one case in which the specimen was too small for pathological diagnosis (Case No. 10), tumor cells were detected in the additional resected specimens in five cases. The detection rate of tumor cells in brain parenchymal lesions was 33.3% (two out of seven cases, three out of nine specimens), whereas all specimens from dural (three cases, four specimens) and bony lesions (two cases, three specimens) were positive for tumor cells. Preoperative imaging, intraoperative findings, and pathological findings of representative cases with fluorescence-positive residual lesions in the brain parenchyma (Figure 1 and Figure 2), bone (Figure 2), and dura mater (Figure 3) are presented in the figures. The final extent of resection, as assessed using postoperative MRI, was the gross total resection in six cases; subtotal resection, five; and partial resection, two. Consequently, fluorescence positivity of the tumor was observed in 11 of the 13 enrolled cases, and additional resection of the pathologically confirmed residual tumor was achieved in five cases, accounting for 38.5% of the total cases.

Adverse events potentially associated with fluorescence diagnosis using 5-ALA included Grade 1 appetite loss and Grade 3 intraoperative hypotension, as classified by CTCAE (Table 3). Intraoperative hypotension occurred from the induction of anesthesia and required the continuous administration of vasopressors until the patient developed anesthesia. However, hypotension did not persist postoperatively.

## 4. Discussion

The results of this prospective clinical study demonstrated that intraoperative fluorescence diagnosis using 5-ALA enabled the detection of additionally resectable tumor lesions that could not be identified under white light microscopy in 38.5% of cases, including those with recurrent intracranial meningiomas and primary intracranial meningiomas suspected of the invasion of surrounding tissues. Notably, tumor cells were pathologically detected in 33.3% of fluorescence-positive lesions within the brain parenchyma, whereas all fluorescence-positive lesions in the bone and dura mater were confirmed to contain tumor cells.

Since Kajimoto et al. first reported intraoperative PpIX fluorescence observations in intracranial meningiomas in 2007, numerous case reports and series have been published regarding intraoperative fluorescence diagnosis using 5-ALA for meningiomas [15,16,17,18,19,20,21,22,23,24,25]. A meta-analysis of 10 studies comprising 206 meningiomas demonstrated that 95% (95% CI: 91.8–97.7%) of tumors exhibited fluorescence positivity [26]. Additionally, studies have reported no significant correlation between PpIX fluorescence intensity and the WHO’s grading of meningiomas [26,27]. Similarly, no correlation was found between the fluorescence intensity and localization of intracranial meningiomas [16]. The reported adverse events associated with 5-ALA fluorescence diagnosis include photosensitivity, transient liver dysfunction, nausea, and vomiting [28].

In our study, fluorescence positivity was observed in 11 out of 13 meningioma cases (84.6%), which is consistent with previously reported fluorescence positivity rates. The two fluorescence-negative cases were both newly diagnosed WHO Grade 1 meningiomas, one located at the skull base and the other in a non–skull base region. As noted above, numerous previous studies have demonstrated that fluorescence positivity in meningiomas does not correlate with histopathological grade. However, another report has indicated that tumor fluorescence intensity may be associated with the expression of key enzymes in the heme biosynthesis pathway, such as ferrochelatase, as well as transmembrane transporters involved in 5-ALA uptake within meningiomas [13]. Unlike gliomas, in which fluorescence positivity is most closely related to tumor grade [29], the factors influencing fluorescence positivity and intensity in meningiomas appear to differ, highlighting the need for further investigation.

Previous studies have highlighted the utility of intraoperative fluorescence diagnosis in meningioma surgery, particularly in detecting tiny remnants that might otherwise be missed using conventional white-light microscopy. However, the extent to which intraoperative fluorescence diagnosis improves the degree of tumor resection compared to white-light surgery has not been thoroughly investigated. To date, the only retrospective study comparing the extent of resection between white-light surgery and fluorescence-guided surgery was conducted by Cornelius et al. [17]. In their analysis of 31 meningioma cases, they reported that, after initial resection under white light, the addition of 5-ALA fluorescence diagnosis enabled further resection in three of 16 WHO Grade 1 meningiomas (19%) and in six of eight WHO Grade 2–3 meningiomas (75%), ultimately improving the extent of resection, particularly in WHO Grade 2–3 meningiomas [17]. Our study is the first prospective investigation to compare the extent of resection between white light and fluorescence-guided surgery. A key feature of our study is that it specifically focused on cases with a high likelihood of residual tumors following surgery, recurrent meningiomas, and primary meningiomas suspected of invading the surrounding tissues based on preoperative imaging. In our study, intraoperative fluorescence diagnosis using 5-ALA allowed additional resection of tumors that were not identified under white light microscopy in 38.5% of all enrolled cases and 45.5% of fluorescence-positive tumor cases. Unlike the study by Cornelius et al., our investigation confirmed the presence or absence of tumor cells in all additional resected specimens via pathological analysis, ensuring a precise assessment of the residual tumor resection rate rather than merely the resection rate of fluorescence-positive lesions. Furthermore, in our study, all five cases in which additional tumor resection was achieved using 5-ALA fluorescence guidance were WHO Grade 1 meningiomas. However, under the WHO 2016 classification and beyond, the two cases in which tumor cells were identified in additional resected brain specimens through fluorescence diagnosis may require reclassification from WHO Grade 1 to Grade 2 atypical meningioma due to suspected brain invasion. Regardless, these findings suggest that intraoperative fluorescence diagnosis is beneficial not only for high-grade meningiomas but also for conventional intracranial meningiomas. Furthermore, although the follow-up period is not yet sufficient, none of the five cases in which additional tumor resection was achieved through intraoperative fluorescence diagnosis have shown recurrence to date. This suggests that fluorescence-guided surgery may offer potential clinical benefits in reducing the recurrence rate.

Among the fluorescence-positive lesions that underwent additional resection guided using intraoperative PpIX fluorescence, tumor cells were pathologically confirmed in all evaluable specimens from bone lesions (two cases, three specimens) and dural lesions (three cases, four specimens), resulting in a 100% positive predictive value. The use of 5-ALA fluorescence diagnosis in the resection of bone-invasive meningiomas has been widely reported. In a retrospective analysis by Della Puppa et al. involving 12 cases of bone-invasive meningiomas, tumor cells were identified in 100% of 57 fluorescence-positive bone specimens [15]. However, among the fluorescence-negative bone specimens, 7 of 41 cases (13%) still harbored tumor invasion, indicating that fluorescence diagnosis does not detect all cases of bone invasion and should be interpreted with caution. Similarly, in a retrospective analysis by Scheichel et al., which included 11 cases of intracranial meningiomas with suspected bone invasion based on preoperative imaging, fluorescence was observed in all bone specimens, and tumor cell infiltration was histologically confirmed in all seven cases where pathological evaluation was performed. Furthermore, they reported fluorescence positivity in soft tissues, including the periosteum (three cases) and temporalis muscle (one case), with pathological confirmation of tumor cell infiltration in all cases [19]. The results of our study, along with these previous findings, suggest that fluorescence-positive lesions that remain after drilling out the bone suspected of tumor invasion under white-light observation may still contain infiltrating tumor cells. Therefore, further resection under fluorescence guidance may be useful for achieving more complete bone-invading tumor resection. The intraoperative fluorescence diagnosis of dural invasive tumors has been previously investigated, particularly the fluorescence properties of the dural tail. However, the significance of dural tail fluorescence remains unclear. Although one study reported that six out of eight cases with preoperatively detected dural tails exhibited fluorescence, with tumor invasion pathologically confirmed in five of these six cases [18], another study analyzing 89 meningioma cases found no fluorescence in any of the dural tails [16]. In our study, dural lesions were not identified in the dural tail but rather at the tumor attachment site in skull base meningiomas, where a residual tumor was detected. After resecting all visible tumor masses under white light, fluorescence observation of the tumor attachment site revealed small fluorescence-positive lesions, prompting additional resectioning of these areas. Although the dural attachment site is typically resected in non-skull base meningiomas, such as convexity meningiomas, in cases of skull base meningiomas, where the dural attachment is often preserved, intraoperative fluorescence diagnosis may facilitate the identification and additional resection of residual tumor lesions, demonstrating its clinical utility.

The significance of fluorescence findings in the adjacent brain parenchyma during intraoperative fluorescence diagnosis using 5-ALA for intracranial meningiomas has not been extensively studied, and a consensus on its interpretation is yet to be established. In a retrospective study by Millesi et al., fluorescence positivity in the adjacent brain parenchyma was observed in 20 of 80 (25%) intracranial meningioma cases; however, these lesions were not histopathologically evaluated [16]. Wilbers et al. reported fluorescence positivity in the adjacent brain parenchyma in a case of recurrent atypical meningioma with tumor cells detected in the corresponding lesion [23]. In a retrospective study, Cornelius et al. observed fluorescence positivity in the adjacent brain parenchyma in eight cases of WHO Grade 2–3 meningiomas, with all cases showing histopathological confirmation of tumor cell infiltration [17]. Wadiura et al. conducted a retrospective histopathological analysis of 191 fluorescence-positive specimens from 85 intracranial meningiomas and reported that, among seven fluorescence-positive brain parenchymal specimens, tumor cells were detected in only one case (14%) [24]. The remaining six fluorescence-positive brain parenchymal specimens exhibited distinct reactive tissue alterations. In our study, tumor cells were detected in only two of seven cases (three of nine specimens), with a detection rate of 33.3% for fluorescence-positive adjacent brain parenchymal lesions. Among the seven cases, six were WHO Grade 1 meningiomas, and one was a WHO Grade 2 atypical meningioma; however, fluorescence-positive brain parenchymal areas in the atypical meningioma case tested negative for tumor cells. Collectively, these findings suggest that fluorescence-positive areas in the adjacent brain parenchyma do not necessarily indicate tumor cell infiltration, and decisions regarding additional resection should be made with caution. If fluorescence positivity is observed in the adjacent brain parenchyma, it is advisable to confirm that the area is not an eloquent region and to submit specimens for rapid intraoperative pathological examination to guide the decision on further resection. One possible explanation for the fluorescence positivity in the brain parenchyma without tumor cell infiltration is the diffusion of PpIX produced within the meningioma into the extracellular fluid, leading to its accumulation in the adjacent brain tissue [30].

This study had several limitations. First, the sample size is relatively small. Second, the intraoperative fluorescence observation devices were not standardized because both microscopes and exoscopes were used. Additionally, the fluorescence intensity was subjectively assessed using a three-tier grading system, which lacks objectivity, as determined by the surgeon. Finally, this study did not evaluate postoperative recurrence or long-term prognosis. To assess fluorescence intensity, quantitative evaluation of PpIX fluorescence using spectroscopic probes and other techniques is being developed, which may contribute to the standardization of fluorescence intensity assessments [25,31,32]. In the future, prospective clinical trials with larger sample sizes are needed to evaluate progression-free survival and overall survival following intracranial meningioma resection guided by intraoperative fluorescence diagnosis. Nevertheless, this study is significant as it is a prospective investigation focusing on invasive meningiomas that clarifies the extent to which intraoperative fluorescence diagnosis enables the additional resection of lesions that would remain undetected under white-light surgery and the identification of the specific sites of tumor invasion where such additional resection is feasible.

## 5. Conclusions

This prospective clinical trial aimed to evaluate the utility of intraoperative fluorescence diagnosis using 5-ALA in improving tumor resection compared to conventional white-light surgery in intracranial meningiomas suspected to invade surrounding tissues. The results demonstrated that intraoperative fluorescence diagnosis enabled the additional resection of residual tumors in 38.5% of all enrolled cases and 45.5% of the fluorescence-positive cases. Although all fluorescence-positive lesions in the bone and dura mater exhibited tumor infiltration, fluorescence positivity in the adjacent brain parenchyma sometimes resulted in false-positive findings. Therefore, the decision to resect fluorescence-positive lesions of the brain parenchyma should be made with caution. Large-scale studies are needed to determine whether intraoperative fluorescence diagnosis using 5-ALA contributes to reduced tumor recurrence and prolonged survival in patients with invasive intracranial meningiomas.

## Figures and Tables

**Table 1:** Summary of patient and tumor characteristics.

	*n* = 13	%
Age, years		
median	66	
range	42–76	
Sex		
male	4	30.8
female	9	69.2
Disease stage		
newly diagnosed	7	53.8
recurrent	6	46.2
Location		
convexity	4	30.8
parasagittal	1	7.7
olfactory groove	1	7.7
planum sphenoidale	1	7.7
sphenoid ridge	2	15.4
spheno-orbital	2	15.4
middle fossa	1	7.7
trigone of the lateral ventricle	1	7.7
Histological diagnosis		
WHO Grade 1	10	76.9
meningothelial	6	46.2
transitional	3	23.1
angiomatous	1	7.7
WHO Grade 2	2	15.4
atypical	2	15.4
WHO Grade 3	1	7.7
anaplastic	1	7.7

**Table 2:** Detailed information of the fluorescence status, identified residual tumor, and additional resection.

Case No.	Location	Histological Diagnosis	Tumor Fluorescence	Residual Tumor First Identified by Fluorescence Diagnosis	Additional Resection Following Fluorescence Diagnosis	Site and Number of Additional Resection		Tumor Cells in Specimens from Additional Resection
1	olfactory groove	meningothelial	None					
2	parasagittal	atypical	Strong	yes	yes	brain	2	no
3	planum sphenoidale	meningothelial	Strong	yes	yes	brain	1	no
						dura	1	yes
4	spheno-orbital	atypical	Vague	no	no			
5	convexity	angiomatous	None					
6	trigone of the lateral ventricle	meningothelial	Strong	yes	yes	brain	1	yes
7	middle fossa	transitional	Strong	yes	yes	brain	1	no
						dura	1	yes
						bone	2	yes
8	convexity	meningothelial	Strong	no	no			
9	convexity	meningothelial	Strong	yes	yes	brain	1	no
10	sphenoid ridge	anaplastic	Strong	yes	yes	dura	2	NA
11	sphen-oorbital	meningothelial	Strong	yes	yes	bone	1	yes
12	convexity	transitional	Vague	yes	yes	brain	1	no
13	sphenoid ridge	transitional	Strong	yes	yes	brain	2	yes

NA: not available.

**Table 3:** Adverse events likely related to 5-ALA.

Adverse Event	Grade 1	Grade 2	Grade 3	Grade 4	Grade 3–4 (%)
Appetite loss	1				0.0
Hypotension			1		7.7

## Data Availability

The data presented in this study are available upon request from the corresponding author.

## Abbreviations

The following abbreviations are used in this manuscript:

5-ALA	5-aminolevulinic acid
MRI	magnetic resonance imaging
PpIX	protoporphyrin IX
WHO	World Health Organization

## References

[1] Ostrom Q.T., Price M., Neff C., Cioffi G., Waite K.A., Kruchko C., Barnholtz-Sloan J.S. (2023). CBTRUS Statistical Report: Primary Brain and Other Central Nervous System Tumors Diagnosed in the United States in 2016–2020. Neuro-Oncology.

[2] Aizer A.A., Bi W.L., Kandola M.S., Lee E.Q., Nayak L., Rinne M.L., Norden A.D., Beroukhim R., Reardon D.A., Wen P.Y. (2015). Extent of resection and overall survival for patients with atypical and malignant meningioma. Cancer.

[3] Winther T.L., Torp S.H. (2017). Significance of the Extent of Resection in Modern Neurosurgical Practice of World Health Organization Grade I Meningiomas. World Neurosurg..

[4] Przybylowski C.J., Suki D., Raza S.M., DeMonte F. (2023). Volumetric extent of resection and survival for recurrent atypical meningioma. J. Neurosurg..

[5] Soni P., Davison M.A., Shao J., Momin A., Lopez D., Angelov L., Barnett G.H., Lee J.H., Mohammadi A.M., Kshettry V.R. (2020). Extent of resection and survival outcomes in World Health Organization grade II meningiomas. J. Neuro-Oncol..

[6] Güdük M., Özduman K., Pamir M.N. (2019). Sphenoid Wing Meningiomas: Surgical Outcomes in a Series of 141 Cases and Proposal of a Scoring System Predicting Extent of Resection. World Neurosurg..

[7] Hénaux P.-L., Bretonnier M., Le Reste P.-J., Morandi X. (2018). Modern Management of Meningiomas Compressing the Optic Nerve: A Systematic Review. World Neurosurg..

[8] Eatz T.A., Eichberg D.G., Lu V.M., Di L., Komotar R.J., Ivan M.E. (2022). Intraoperative 5-ALA fluorescence-guided resection of high-grade glioma leads to greater extent of resection with better outcomes: A systematic review. J. Neuro-Oncol..

[9] Stummer W., Pichlmeier U., Meinel T., Wiestler O.D., Zanella F., Reulen H.-J., ALA-Glioma Study Group (2006). Fluorescence-guided surgery with 5-aminolevulinic acid for resection of malignant glioma: A randomised controlled multicentre phase III trial. Lancet Oncol..

[10] McCracken D.J., Schupper A.J., Lakomkin N., Malcolm J., Bray D.P., Hadjipanayis C.G. (2022). Turning on the light for brain tumor surgery: A 5-aminolevulinic acid story. Neuro-Oncology.

[11] Barbosa B.J.A.P., Mariano E.D., Batista C.M., Marie S.K.N., Teixeira M.J., Pereira C.U., Tatagiba M.S., Lepski G.A. (2014). Intraoperative assistive technologies and extent of resection in glioma surgery: A systematic review of prospective controlled studies. Neurosurg. Rev..

[12] Specchia F.M.C., Monticelli M., Zeppa P., Bianconi A., Zenga F., Altieri R., Pugliese B., Di Perna G., Cofano F., Tartara F. (2021). Let Me See: Correlation between 5-ALA Fluorescence and Molecular Pathways in Glioblastoma: A Single Center Experience. Brain Sci..

[13] Spille D.C., Bunk E.C., Thomas C., Özdemir Z., Wagner A., Akkurt B.H., Mannil M., Paulus W., Grauer O.M., Stummer W. (2023). Protoporphyrin IX (PpIX) Fluorescence during Meningioma Surgery: Correlations with Histological Findings and Expression of Heme Pathway Molecules. Cancers.

[14] Bunk E.C., Wagner A., Stummer W., Senner V., Brokinkel B. (2021). 5-ALA kinetics in meningiomas: Analysis of tumor fluorescence and PpIX metabolism in vitro and comparative analyses with high-grade gliomas. J. Neuro-Oncol..

[15] Della Puppa A., Rustemi O., Gioffrè G., Troncon I., Lombardi G., Rolma G., Sergi M., Munari M., Cecchin D., Gardiman M.P. (2014). Predictive value of intraoperative 5-aminolevulinic acid–induced fluorescence for detecting bone invasion in meningioma surgery. J. Neurosurg..

[16] Millesi M., Kiesel B., Mischkulnig M., Martínez-Moreno M., Wöhrer A., Wolfsberger S., Knosp E., Widhalm G. (2016). Analysis of the surgical benefits of 5-ALA–induced fluorescence in intracranial meningiomas: Experience in 204 meningiomas. J. Neurosurg..

[17] Cornelius J., Slotty P., Kamp M., Schneiderhan T., Steiger H., El-Khatib M. (2014). Impact of 5-aminolevulinic acid fluorescence-guided surgery on the extent of resection of meningiomas–With special regard to high-grade tumors. Photodiagnosis Photodyn. Ther..

[18] Kajimoto Y., Kuroiwa T., Miyatake S.-I., Ichioka T., Miyashita M., Tanaka H., Tsuji M. (2007). Use of 5-aminolevulinic acid in fluorescence-guided resection of meningioma with high risk of recurrence. J. Neurosurg..

[19] Scheichel F., Popadic B., Kitzwoegerer M., Ungersboeck K., Marhold F. (2019). Fluorescence-guided resection in bone and soft tissue infiltrating meningiomas. Acta Neurochir..

[20] Coluccia D., Fandino J., Fujioka M., Cordovi S., Muroi C., Landolt H. (2010). Intraoperative 5-aminolevulinic-acid-induced fluorescence in meningiomas. Acta Neurochir..

[21] Potapov A.A., Goryaynov S.A., Okhlopkov V.A., Shishkina L.V., Loschenov V.B., Savelieva T.A., Golbin D.A., Chumakova A.P., Goldberg M.F., Varyukhina M.D. (2016). Laser biospectroscopy and 5-ALA fluorescence navigation as a helpful tool in the meningioma resection. Neurosurg. Rev..

[22] Marbacher S., Klinger E., Schwyzer L., Fischer I., Nevzati E., Diepers M., Roelcke U., Fathi A.-R., Coluccia D., Fandino J. (2014). Use of fluorescence to guide resection or biopsy of primary brain tumors and brain metastases. Neurosurg. Focus.

[23] Wilbers E., Hargus G., Wölfer J., Stummer W. (2014). Usefulness of 5-ALA (Gliolan®)-derived PPX fluorescence for demonstrating the extent of infiltration in atypical meningiomas. Acta Neurochir..

[24] Wadiura L.I., Millesi M., Makolli J., Wais J., Kiesel B., Mischkulnig M., Mercea P.A., Roetzer T., Knosp E., Rössler K. (2020). High Diagnostic Accuracy of Visible 5-ALA Fluorescence in Meningioma Surgery According to Histopathological Analysis of Tumor Bulk and Peritumoral Tissue. Lasers Surg. Med..

[25] Valdes P.A., Millesi M., Widhalm G., Roberts D.W. (2019). 5-aminolevulinic acid induced protoporphyrin IX (ALA-PpIX) fluorescence guidance in meningioma surgery. J. Neuro-Oncol..

[26] Foster N., Eljamel S. (2016). ALA-induced fluorescence image guided surgery of meningiomas: A meta-analyses. Photodiagnosis Photodyn. Ther..

[27] Dijkstra B.M., Jeltema H.-R., Kruijff S., Groen R.J.M. (2018). The application of fluorescence techniques in meningioma surgery—A review. Neurosurg. Rev..

[28] Chung I.W.H., Eljamel S. (2013). Risk factors for developing oral 5-aminolevulenic acid-induced side effects in patients undergoing fluorescence guided resection. Photodiagnosis Photodyn. Ther..

[29] Bianconi A., Bonada M., Zeppa P., Colonna S., Tartara F., Melcarne A., Garbossa D., Cofano F. (2023). How Reliable Is Fluorescence-Guided Surgery in Low-Grade Gliomas? A Systematic Review Concerning Different Fluorophores. Cancers.

[30] Masubuchi T., Kajimoto Y., Kawabata S., Nonoguchi N., Fujishiro T., Miyatake S.-I., Kuroiwa T. (2013). Experimental Study to Understand Nonspecific Protoporphyrin IX Fluorescence in Brain Tissues Near Tumors After 5-Aminolevulinic Acid Administration. Photomed. Laser Surg..

[31] Knipps J., Beseoglu K., Kamp M., Fischer I., Felsberg J., Neumann L.M., Steiger H.-J., Cornelius J.F. (2017). Fluorescence Behavior and Dural Infiltration of Meningioma Analyzed by 5-Aminolevulinic Acid–Based Fluorescence: Operating Microscope Versus Mini-Spectrometer. World Neurosurg..

[32] Cornelius J., Placke J., Knipps J., Fischer I., Kamp M., Steiger H. (2016). Minispectrometer with handheld probe for 5-ALA based fluorescence-guided surgery of brain tumors: Preliminary study for clinical applications. Photodiagnosis Photodyn. Ther..

